# Prostate embolization in the treatment of benign prostatic hyperplasia: what’s the point?

**DOI:** 10.1590/0100-3984.2021.54.4e1

**Published:** 2021

**Authors:** Thiago Franchi Nunes, Gustavo Pipoca Andrade

**Affiliations:** 1 Interventional radiologist at the Hospital Universitário Maria Aparecida Pedrossian da Universidade Federal de Mato Grosso do Sul (HUMAP-UFMS), Campo Grande, MS, Brazil. E-mail: thiagofranchinunes@gmail.com.; 2 Interventional radiologist for Angiorad, Recife, PE, Brazil. E-mail: guga.andrade@gmail.com.

Benign prostatic hyperplasia (BPH) is common among elderly men, approximately 50% of those over 60 years of age showing symptoms of the condition^(1,2)^. The main clinical manifestations of BPH are usually grouped together as obstructive lower urinary tract symptoms, including decreased urinary flow, nocturia, urinary urgency, splitting of the urinary stream, and urinary hesitancy. The symptoms are graded with an internationally accepted questionnaire, the International Prostate Symptom Score.

The therapeutic approach to BPH depends on the symptom score, the mildest cases being followed clinically. Moderate cases should be treated pharmacologically, with a single-drug regimen or drug combination therapy^(3)^. In cases of refractory disease or those in which the pharmacological treatment cannot be continued, invasive options should be considered; the standard options are currently transurethral resection of the prostate and open prostatectomy, the choice between the two depending on the prostate volume^(4,5)^. Within the urology community, numerous alternatives to those standard options have emerged in the last decade and continue to emerge, indicating the need for less aggressive therapies. The current therapeutic arsenal comprises ablative therapies that are thermal (with heat or cold) or chemical (with Botox or alcohol), as well as vaporization, implantation of a UroLift device, enucleation of the prostate with a greenlight or holmium laser, and prostatic artery embolization (PAE). This search for less aggressive options is not aimless; it is due to the fact that the standard techniques can have complications and adverse effects, such as retrograde ejaculation, sexual dysfunction, urinary incontinence, hemorrhagic complications, and urethral stricture^(6,7)^.

Recently, PAE emerged as a minimally invasive alternative, performed by interventional radiology, for the treatment of obstructive lower urinary tract symptoms secondary to BPH^(8)^. In PAE, microspheres are injected directly into the prostatic arteries bilaterally (or unilaterally, when there is technical difficulty) to promote arterial obstruction and parenchymal ischemia, thus reducing prostate volume and symptom severity^(9)^. Arterial embolization of the prostate was first reported by Mitchell et al.^(10)^, who employed it to control severe prostatic hemorrhage. After the safety of PAE had been proven, in the first half of the 2010s, numerous studies demonstrated its short- and long-term effectiveness, more than a dozen meta-analyses or systematic reviews, all of which showed the safety and efficacy of the technique, having been published in the last five years^(11-14)^.

The article authored by Assis et al.^(15)^, published in this issue of Radiologia Brasileira, again confirms the safety and efficacy of PAE in patients with very large prostates (≥ 200 g). They performed a retrospective study of 18 patients, showing that the rate of technical and clinical success of PAE was over 94%, even in giant prostates, for which the degree of surgical difficulty is greater. This is a significant finding, given that transvesical prostatectomy continues to be the surgical standard of care, despite the fact that it is associated with considerable morbidity. It is of note that PAE is no longer a complex procedure and that the size of the prostate no longer precludes its performance. In fact, compensatory hypertrophy of the artery tends to facilitate the technique even further. Therefore, embolization should be considered as an option for individuals with BPH, either as a definitive treatment (for patients who are not candidates for surgery) or as a down-staging strategy prior to less aggressive procedures (i.e., for smaller prostates). One limitation of the Assis et al.^(15)^ study was that, although the authors evaluated procedures performed since 2013, they reported follow-up periods of only three months.

In conclusion, the huge number of new alternative treatments for BPH that have emerged and continue to emerge are indicative of the difficulty related to and dissatisfaction with the treatments that are considered the gold standards: transurethral resection of the prostate and transvesical prostatectomy. The safety, efficacy, and cost-effectiveness of PAE have been proven, its advantages and disadvantages now being well known. During this time of pandemic and extensive discussion of scientific evidence, which has led to a blurring of the division between politics and science, PAE already has all the prerequisites to become an option in the therapeutic arsenal against BPH. What’s lacking: the political will or the scientific wherewithal?
